# Effects of micronized bamboo powder on growth performance, serum biochemical indexes, cecal chyme microflora and metabolism of broilers aged 1–22 days

**DOI:** 10.1007/s11250-022-03172-0

**Published:** 2022-04-18

**Authors:** Fawen Dai, Tao Lin, Lumin Cheng, Jian Wang, Jianjun Zuo, Dingyuan Feng

**Affiliations:** 1 Present Address: College of Life Science, Leshan Normal University, No. 778, Binghe Road, Leshan, Sichuan 614000 People’s Republic of China; 2 Guang’an Feed Industry Management Office, Guang’an, Sichuan 638000 People’s Republic of China; 3 Sichuan Juxing Group, Sichuan, Chengdu, Sichuan 610095 People’s Republic of China; 4 College of Animal Science, South China Agricultural University, Guangzhou, Guangdong 510642 People’s Republic of China

**Keywords:** Fiber, Broilers, Short chain fatty acids, Intestinal microflora, Metabolism pathways

## Abstract

Adding insoluble fiber to diet of broilers has been reported to improve intestinal health and promote growth performance. Bamboo powder is a cheap raw material with rich insoluble fiber. This study aims to explore the effects of feeding micronized bamboo powder (MBP) on growth performance, serum biochemical indexes, intestinal microflora, and metabolism of broilers. A total of 1440 1-day-old slow-growing Ephedra chickens were randomly divided into three groups considering gender and body weight: (1) Group D: feeding with basal diet without antibiotics; (2) Group E: feeding with basal diet supplemented with 5% rice bran (RB); (3) Group F: feeding with basal diet supplemented with 1% MBP. Each group involved 8 replicates feeding for 22 days, with 60 chickens per replicate. Various indexes were detected. For the growth performance, the weight gain and feed consumption ratio (G: F) of Group F supplemented with MBP is 0.57 ± 0.04, which is significantly higher than that of E group supplemented with RB (0.52 ± 0.01, *P* < 0.05). For the serum biochemical indexes, the glutathione peroxidase activity in Group F is significantly higher than that of Group D, while the malondialdehyde content is significantly lower than that of Group D and Group E (*P* < 0.05 for all). The fresh cecal chyme is taken for determination. In Group F, the α diversity index Faith_pd is significantly lower in Group F than that of Group D. The microorganism species in cecal chyme of Group F and Group E are also different. The metabolic pathways of Group F, mainly in fatty acid metabolism, amino acid metabolism and intestinal immune IgA production, were different from those of Group D and Group E. Adding 1% MBP to broiler diet can enhance the anti-oxidant capacity, improve chyme microflora, regulate the metabolism pathways responsible for intestinal fatty acids, amino acids, and immunity.

## Introduction

With the rapid development of animal husbandry, the reduced resource and rising cost of feed have become the critical factors for limiting its green and lasting development in the future. The exploration of unconventional feed resources is urgently needed (Makkar 2018). Under the background of “Prohibiting the addition of growth-promoting antibiotics in feed” in China, adding appropriate fiber may be one of the most effective alternatives to improve animal intestinal health and growth performance (Jiménez-Moreno et al. 2016; Shang et al. 2021). For instance, adding an appropriate amount of IDF in broiler diets can decrease the moisture content of the bedding (Kheravii et al. 2017), stimulate the development of chicken stomach (Donadelli et al. 2019), reduce fat deposition (Nassar et al. 2019), lower the pH of muscle stomach, improve nutrient digestibility (Jiménez-Moreno et al. 2019) and utilization rate (Nassar et al. 2019), so as to promote the growth of chicken (Donadelli et al. 2019). Adding 0.5, 1, and 2 kg/t processed lignin significantly promoted the development of jejunum villi (increased by 22.0%, 40.7%, and 34.8%, respectively) and crypt, and the apparent surface area of villi increased by 11.7%, 30.5%, and 1.2%, respectively. The highest growth rate of villi was in 1 kg group (Sozcu 2019). However, the effects are different for various fibers in broiler feed (Jiménez-Moreno et al. 2009; Jiménez-Moreno et al. 2019). Therefore, it is very important to find suitable source of fibers.

Bamboo is one of the renewable natural resources with the fastest growth rate and highest yield. The bamboo resources are abundant with wide distribution (He et al. 2014; Lancefield et al. 2017). When pandas fed with whole bamboo, the apparent digestibility of crude protein and crude fiber is 33.8% and 31.8% (Sims et al. 2007). Bamboo powder is rich in insoluble dietary fiber (IDF) (Felisberto et al. 2017), leaf flavonoids and polysaccharides, which is conductive to regulating the immunity and antioxidant capacity of animals (Ge et al. 2020). Bamboo shoot shell fibers exhibit strong cholesterol adsorption activity and prebiotic potential, which can promote the growth of lactic acid bacteria and increase the fermentability of substrates (Wu et al. 2020). It can also improve the disorder of fat metabolism in mice with hyperlipidemia (Luo et al. 2017). Thus, it is expected to become a source of starch and fiber in feed. At present, bamboo-based fiber materials have been added to pig feed and sheep feed (Okano et al. 2009; Oguri et al. 2013), but there has been no report on broiler feed.

In traditional opinions, adding fiber to broiler feed may dilute nutrition, thus making negative effects on feed digestibility and growth performance (Aftab and Bedford 2018). However, existing research show that, moderate content of IDF can lower the moisture content of litter (Kheravii et al. 2017), reduce fat deposition and increase the growth rate (Nassar et al. 2019), and stimulate the development of chicken stomach, thus improving the nutrient utilization rate and promoting the growth of chicken (Donadelli et al. 2019). Similar results can be obtained by supplementing an appropriate level of structural IDF raw materials to the low-fiber diet of broilers (Jiménez-Moreno et al. 2016).

The processing technology and fiber resource can also change the chemical composition and nutritional composition of fibers (Donadelli et al. 2019). Micronization can change the particle size distribution and functional characteristics of IDF in rice bran (RB), thus affecting the digestibility of raw materials. The micronized RB show high water-holding capacity, swelling capacity, high phenol extraction rate and oxidation resistance (Zhao et al. 2018). The bionic digestion experiment performed on wheat show that the in vitro digestibility of both dry matter and crude protein is increased with decreased grain size (Bao et al. 2017). The decreased grain size can improve total energy, dry matter, total fiber and IDF apparent ileal digestibility. There are also significant interactions between grain size and fiber source, thus affecting the digestibility of hindgut fiber (Zhao et al. 2019). The fiber resource can also make effects. Compared with the fiber extracted from bamboo shoot shell, the feed bamboo powder made from bamboo stems contains a certain amount of lignin. Ultra-micronization can increase the content of soluble fiber components, reduce the lignin level, and improve the water holding capacity and fat adsorption of raw materials (Speroni et al. 2020).

The previous studies of our group found that the D_90_ (the cumulative particle size distribution number is 90%) of micronized bamboo powder (MBP) has been lower than that of conventional bamboo powder(Dai et al. 2021). MBP exhibited more obvious growth promoting effects in weaning piglets. In this study, MBP has been added to broiler diet and fed for 22 days. Rice bran (RB) is rich in CF and NDF and often used as feed in Chinese broiler production, but its fat is high and easy to oxidize. Then, we explore the effects of high NDF diet with 1% MBP or 5% RB on growth performance, serum nutrition physiology and anti-oxidation, digestive organ index, cecal chyme volatile fatty acids, cecal chyme microflora structure and metabolic changes of broilers. We hope the results of this study can provide data and information for the applications of MBP in broiler diet.

## Material and methods

### The preparation of MBP

Bamboo poles (5–6 years old, produced from Sichuan, China) were applied. After the removal of the bamboo outer skin (Huang et al. 2019), the bamboo poles were preliminarily crushed by a cutter crusher (Model 600, Zhengzhou Chuangyi Machinery Equipment Co., Ltd.) and dried until the water content was 10–12%. The bamboo powder was further crushed by an impact mill (ZJ-C100, Sichuan Zhongjin Powder Equipment Co., Ltd.) and then passed through a 200-mesh sieve. The particle size distribution of MBP was determined by Laser particle size distribution analyzer (BT-9300ST, Dandong Bettersize Instrument Co., LTD, China): D_98_ (the cumulative particle size distribution number is 98%) was 93.33 μm and D_90_ was 55.47 μm. The content of moisture, CP, ash was tested according to Wang’s method, which was 7.49, 1.63, 1.0, respectively (Wang et al. 2018). The content of acid detergent fiber (ADF) and neutral detergent fiber (NDF) was determined with reference method (Van Soest et al. 1991), which was 65.80% and 82.94%, respectively.

### Animals and groups

A total of 1440 slow-growing ephedra chickens (1 day old, body weight: 35.16 ± 0.52 g, Jinling, an improved local breed) were obtained from a commercial hatchery (WEN’S Group, China). Birds were randomly divided into three groups considering gender and body weight (BW): (1) Group D: feeding with basal diet without antibiotic; (2) Group E: feeding with basal diet supplemented with 5% RB; (3) Group F: feeding with basal diet supplemented with 1% MBP. There are 8 replicates (half male and half female in one replicate) in each group, 60 chickens in each replicate, and the chickens in one replicate were fed in one coop. The chickens were cultured with ground bedding and fed according to the routine feeding procedure. They were immunized normally, free feeding and drinking. The chickens were fed for 22 days.

### Diet composition and nutrition level

The basal diet was prepared according to the nutrition requirement standard (NY/T3645-2020 Nutrient requirements of yellow chickens). The normal nutrition index of bamboo powder was determined and ME is predicted according to our previous study of MBP in weaned piglets (Dai et al. 2021). Table 1 shows the ingredient and nutrient level in the experimental diets, the content of fiber and NDF in group E and F is higher than that of group D.

**Table 1:** Composition and nutrition level of the diet in three groups (as-fed basis, g/kg)

Items	Group D (Basal diet)	Group E (Basal diet with 5% RB)	Group F (Basal diet with 1% MBP)
Corn	640.1	594.1	630.1
Corn gluten meal	20	20	20
Soybean oil	4	7	4
Extruded soybean	20	20	20
Fermented soybean meal	30	30	30
Soybean meal (CP46%)	187	180	187
RB	-	50	-
MBP	-	-	10
Cottonseed meal (CP46%)	50	50	50
Premix*	48.9	48.9	48.9
Total	1000	1000	1000
Calculated composition			
ME (Kcal/kg)	2890	2890	2890
CP (%)	19.5	19.5	19.5
EE (%)	3.2	4.1	3.2
CF (%)	3.0	3.3	3.4
NDF (%)	9.9	10.5	10.6
Lys (%)	1.12	1.12	1.12
Met (%)	0.52	0.52	0.52
Thr (%)	0.79	0.79	0.79
Ca (%)	0.96	0.96	0.96
AP (%)	0.48	0.48	0.48

^*^ Premix supplies the following nutrients (per kilogram of diet): vitamin A (retinyl acetate), 10,000 IU; vitamin D3 (cholecalciferol), 2500 IU; vitamin E (dl-α-tocopheryl acetate), 40 IU; vitamin K3 (menadione sodium bisulfite), 2 mg; vitamin B1 (thiamine), 2 mg; vitamin B2 (riboflavin), 6 mg; vitamin B3 (niacin), 50 mg; vitamin B5 (calcium d-pantothenate), 12 mg; vitamin B6 (pyridoxine hydrochloride), 5 mg; vitamin B12 (cyanocobalamin), 0.02 mg; biotin, 0.12 mg; folic acid, 1.5 mg; choline chloride, 600 mg; Mn(MnSO_4_∙H_2_O), 80 mg; Fe(FeSO_4_∙7H_2_O), 80 mg; Zn (sulfate and oxide) 60 mg; Cu(CuSO_4_∙5H_2_O), 8 mg; I (iodide), 1 mg; Se(Na_2_SeO_3_), 0.3 mg; Co (cobalt), 0.3 mg; NaCl, 3 g; antioxidant, 40 mg

### Sample collection

On the 22^nd^ day, 2 broilers (one male and the other female) who had been fasting for 12 h were randomly selected and weighed in each repeat of three groups. The venous blood was collected and centrifuged at 3000 rpm for 10 min. The obtained serum samples were stored at − 80 ℃. Then, the broilers were sacrificed by cervical dislocation. The cecal chyme was collected and stored in a sterile centrifuge tube, frozen with liquid nitrogen, and stored at − 80 ℃ for further tests.

### Indexes and measurements

Growth performance: The broilers in each repeat (in the same coop) of three groups were weighed at the beginning and end of the experiments. The daily feed intake was recorded for calculating the average daily gain (ADG) and average daily feed intake (ADFI). The ratio of gain to feed consumption (G: F) was calculated as: ADG/ADFI. Organ index: After the broilers were sacrificed, the heart, liver, spleen, bursa of fabricius, muscular stomach and glandular stomach were taken. The contents of muscular stomach and glandular stomach were removed. All the organs were rinsed. Excess water and blood were absorbed with filter paper, excess tissue and fat were removed. Then, the organs were weighed. The duodenum, jejunum, ileum, and colorectal were separated, and the length of each intestine was measured. Organ index (%) = weight of organ/weight of alive broilers × 100%. Intestinal organ index (cm/g) = length of intestine/weight of alive broilers.

Serum biochemical indexes: Enzyme-linked immunosorbent assay (ELISA) was used to quantify the serum physiological metabolism indexes (including glucose, cholesterol, triglyceride, total protein, and urea nitrogen), and serum antioxidant indicators (malondialdehyde, catalase, glutathione peroxidase and superoxide dismutase). ELISA kits were purchased from Nanjing Jiancheng Institute of Bioengineering (Jiangsu, China).

Volatile fatty acids in cecal chyme: The volatile fatty acids in collected cecal chyme were determined with gas-chromatography (GC) according to the method reported by Zhou et al. (2014). Agilent HP-INNOWAX capillary column gas chromatograph (TRACE 1310-ISQ LT, Thermo, USA; Capillary column: 30 m*0.25 mm ID*0.25 μm) was involved.

The cecal chyme microflora: The genome DNA of cecal chyme microflora was extracted with DNA extraction kit (QIAGEN, Inc., Netherlands) and quantified with Nanodrop (thermo fisher scientific Inc., USA). The primers were designed according to the V3-V4 conserved region of microbial 16SrRNA sequence (the upstream primer is 5′-ACTCCTACGGGAGGCAGCA-3′, the downstream primer is 5′-GGACTACHVGGGTWTCTAAT-3′). The PCR amplification was performed with Pfu high fidelity DNA polymerase from TransGen Biotech.Co., Ltd. (Beijing, China). PCR amplification products were purified by adding magnetic beads (Vazyme VAHTSTM DNA Clean Beads). The amplification products were analyzed with Illumina MiSeq6000 platform for Paired-end sequencing by Suzhou Panomik Biomedical Technology Co., Ltd. (Jiangsu, China).

According to the analysis flow of qiime 2 (2019.4) DADA2 method, sequence denoising is performed (Callahan et al. 2016), and sequence OTU clustering is performed by the analysis flow of Vsearch software (Rognes et al. 2016). Greengenes database (Release 13.8, http://greengenes.secondgenome.com/), Silva database (Release132), UNITE database (Release 8.0, https://unite.ut.ee/), and nt database (ftp://ftp.ncbi.nih.gov/blast/db/) were included. The microbial species were annotated with QIIME2 classify-sklearn algorithm and BROCC algorithm.

The species composition was analyzed. The ASV/OUT table was flattened by the rarefaction method, and the depth of flattening was set to 95% of the minimum sample sequence. By analyzing and counting the flattened ASV/OTU table, the specific composition of microbial community in each sample at different classification levels was obtained. The relative abundance composition of different experimental groups at phylum and genus level was compared. The α diversity was carried out, in which Chao1 characterized the abundance and Faith_pd indicated the diversity of evolution.

The cecal chyme metabolomics: 100 mg (± 1%) of sample was accurately weighed and placed in a 2 mL EP tube. 0.6 mL of 2-chlorophenylalanine in methanol (4 ppm, − 20 ℃) was added and vortex for 30 s. After adding 100 mg of glass beads, the solution was ground for 90 s at 60 Hz in an issue grinder, and then ultrasound for 10 min at room temperature. After centrifuging at 12,000 rpm at for 10 min, 300 μL of supernatant was filtered by 0.22 μm membrane. The filtrate was added to the detection bottle. Take 20 µL from each sample to be tested and mix them into QC samples. The remaining samples were applied for LC–MS detection.

Chromatography analysis: The chromatography was performed with Thermo Ultimate 3000 and the ACQUITY UPLC® HSS T3 1.8 µm (2.1 × 150 mm) column. Autosampler was set at 8 ℃, flow rate was 0.25 mL/min, column temperature was 40 ℃. 2 μL of sample was injected for gradient elution. The mobile phase: cation was 0.1% formic acid water (A)-0.1% formic acid acetonitrile (B); the anion was 5 mM ammonium formate water (C)-acetonitrile (D). Gradient elution procedure was as follows: 0–1 min, 2% B/D; 1–9 min, 2–50% B/D; 9–12 min, 50–98% B/D; 12–13.5 min, 98% B/D; 13.5–14 min, 98–2% B/D; 14–20 min, 2% B-positive mode (14–17 min, 2% D-negative mode).

Mass spectrometry (MS) analysis: The MS analysis was performed with Thermo Q Exactive HF-X, electrospray ion source (ESI), positive and negative ion ionization modes. The positive ion spray voltage was 3.50 kV and the negative ion spray voltage was 2.50 kV. Sheath gas was 30 arb, and auxiliary gas was 10 arb. The capillary temperature was 325 ℃. The scanning was performed with resolution of 60,000 and the range of 81–1,000. HCD was used for secondary cracking and the collision voltage was 30 eV.

LC–MS data processing and differential metabolite identification: peaks identification, peak filtering and peaks alignment were performed with XCMS package of R (v3.3.2). The obtained information included mass to charge ratio (m/z), retention time, and peak area (m/z). The differential metabolites were identified according to the following conditions: *Mann–Whitney-Wilcoxon* Test was applied for statistical comparison between the two groups, and the screening condition was OPLS-DA first principal component variable importance value projection (VIP) > 1 and *P* value < 0.5; *Kruskal–Wallis* Test was applied for the statistical comparison among multiple groups, and the screening condition was VIP > 1 and *P* value < 0.5.

The accurate molecular weight of the metabolite (error < 30 ppm) was obtained. Then, the fragment information obtained according to the MS/MS mode was retrieved in the Human Metabolome Database (HMDB) (http://www.hmdb.ca), Metlin (http://metlin.scripps.edu), Massbank (http://www.massbank.jp/), LipidMaps (http://www.lipidmaps.org), mzclound (https://www.mzcloud.org). The information was matched and annotated to obtain accurate information of metabolites.

### Data processing and statistical analysis

Data were expressed as Mean ± Standard deviation (SD, n = 8). SPSS 25.0 was applied for statistical analysis. One-way analysis of variance (ANOVA) and independent sample *T-test* was applied for analyzing the differences of growth performance, serum physiological and biochemical indexes, organ indexes, volatile fatty acids, and microflora diversity index. *P* < 0.05 indicated that the difference was significant, and *P* < 0.10 indicated a tendency of difference.

Biomarkers of cecal chyme microbial differences were analyzed with Linear discriminant analysis (LDA) Effect Size, the nonparametric *Kruskal–Wallis* and *Wilcoxon* rank sum test.

## Results

### The effects of MBP on growth performance of broilers

**Fig. 1:**
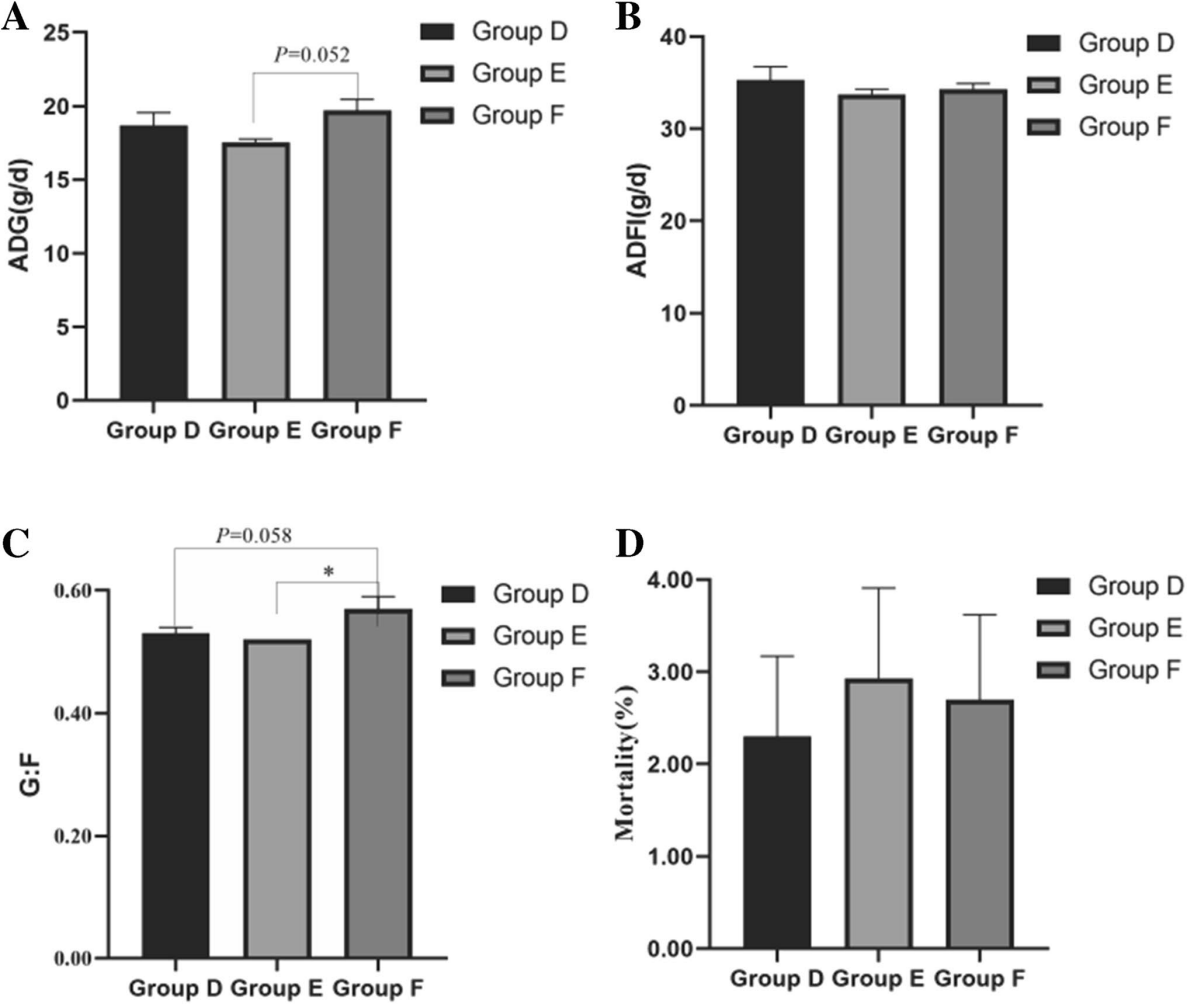
Effects of dietary fibers on growth performance of broilers. (**A**) ADG, (**B**) ADFI, (**C**) G: F, (**D**) Mortality. Group D: Control group, antibiotic-free basal diet; Group E: basal diet + 5% RB; Group F: basal diet + 1% MBP. Data were presented as Mean ± SD (n = 8). Statistical trend at *P* = 0.05–0.10, “*” indicated *P* < 0.05

The initial average BW of broilers in Group D, E and F was 35.31 ± 0.71, 35.42 ± 0.34 and 35.00 ± 0.48, respectively, without significant difference (*P* > 0.05), which was shown in Table 2. After feeding for 22 days, the final average BW was 483.59 ± 42.17, 455.81 ± 12.06 and 507.09 ± 36.06, respectively, without significant difference (*P* > 0.05). The effects of dietary fibers on growth performance of broilers are shown (Fig. 1). The ADG of broilers in Group F tends to be higher than that of Group E (*P* = 0.052, Fig. 1A), and no difference was detected between Group F and Group D (*P* > 0.05). There is no significant difference in ADFI of three groups (*P* > 0.05, Fig. 1B). The G: F of broilers in Group F is 0.57 ± 0.04, which is significantly higher than that of Group E (0.52 ± 0.01, *P* < 0.05, Fig. 1C) and tends to be higher than that of Group D (0.53 ± 0.03, *P* = 0.058). The mortality is not significantly varied in three groups (*P* > 0.05, Fig. 1D). In general, Group F with 1% MBP performed best ADG and G:F, although the difference between Group F and Group D was not significant (*P* > 0.05).

**Table 2:** Effect of dietary fiber on growth performance and mortality rate of broilers

Items	Group D (Basal diet)	Group E (Basal diet with 5% RB)	Group F (Basal diet with 1% MBP)
IW/g	35.31 ± 0.71	35.42 ± 0.34	35.00 ± 0.48
FW/g	483.59 ± 42.17	455.81 ± 12.06	507.09 ± 36.06
ADG/g	18.68 ± 1.73	17.52 ± 0.51	19.67 ± 1.51
FDI/g	35.32 ± 2.83	33.74 ± 1.13	34.31 ± 1.20
G:F	0.53 ± 0.01^ab^	0.52 ± 0.00^a^	0.57 ± 0.02^b^
Mortality rate/%	2.30 ± 0.87	2.93 ± 0.98	2.70 ± 0.92

Different superscript letters indicate significant difference (*P* < 0.05). Data are presented as Mean ± SD (n = 8)

### The effects of MBP on organ indexes of broilers

The effects of dietary fibers on organ indexes of broilers are shown (Table 3). There is no significant difference in organ indexes of heart, liver, spleen, bursa of fabricius, muscular stomach, glandular stomach, jejunum, ileum and colorectal among the three groups (*P* > 0.05 for all). The index of glandular stomach in Group E tends to be higher than that of Group D (*P* = 0.060). The index of duodenum is significantly higher than that of Group F (*P* = 0.003), and it tends to be higher than that of Group D (*P* = 0.104).

**Table 3:** Effect of dietary fiber on organ indexes of broilers

Items	Group D (Basal diet)	Group E (Basal diet with 5% RB)	Group F (Basal diet with 1% MBP)
Heart/%	0.64 ± 0.03	0.62 ± 0.02	0.63 ± 0.03
Liver/%	2.98 ± 0.07	3.07 ± 0.07	2.95 ± 0.04
Spleen/%	0.15 ± 0.01	0.16 ± 0.01	0.16 ± 0.01
Bursa of fabricius/%	0.40 ± 0.03	0.39 ± 0.02	0.36 ± 0.01
Gizzard/%	2.86 ± 0.10	2.72 ± 0.16	2.82 ± 0.08
Glandular stomach/%	0.56 ± 0.02	0.61 ± 0.02	0.57 ± 0.02
Duodenum, cm/gBW	0.044 ± 0.002^ab^	0.047 ± 0.001^a^	0.042 ± 0.001^b^
Jejunum, cm/gBW	0.104 ± 0.005	0.106 ± 0.003	0.097 ± 0.004
Ileum, cm/gBW	0.099 ± 0.006	0.104 ± 0.003	0.100 ± 0.004
Colorectal, cm/gBW	0.013 ± 0.001	0.014 ± 0.001	0.013 ± 0.001

Different superscript letters indicate significant difference (*P* < 0.05). Data are presented as Mean ± SD (n = 8)

### The effects of MBP on serum biochemical indicators of broilers

**Fig. 2:**
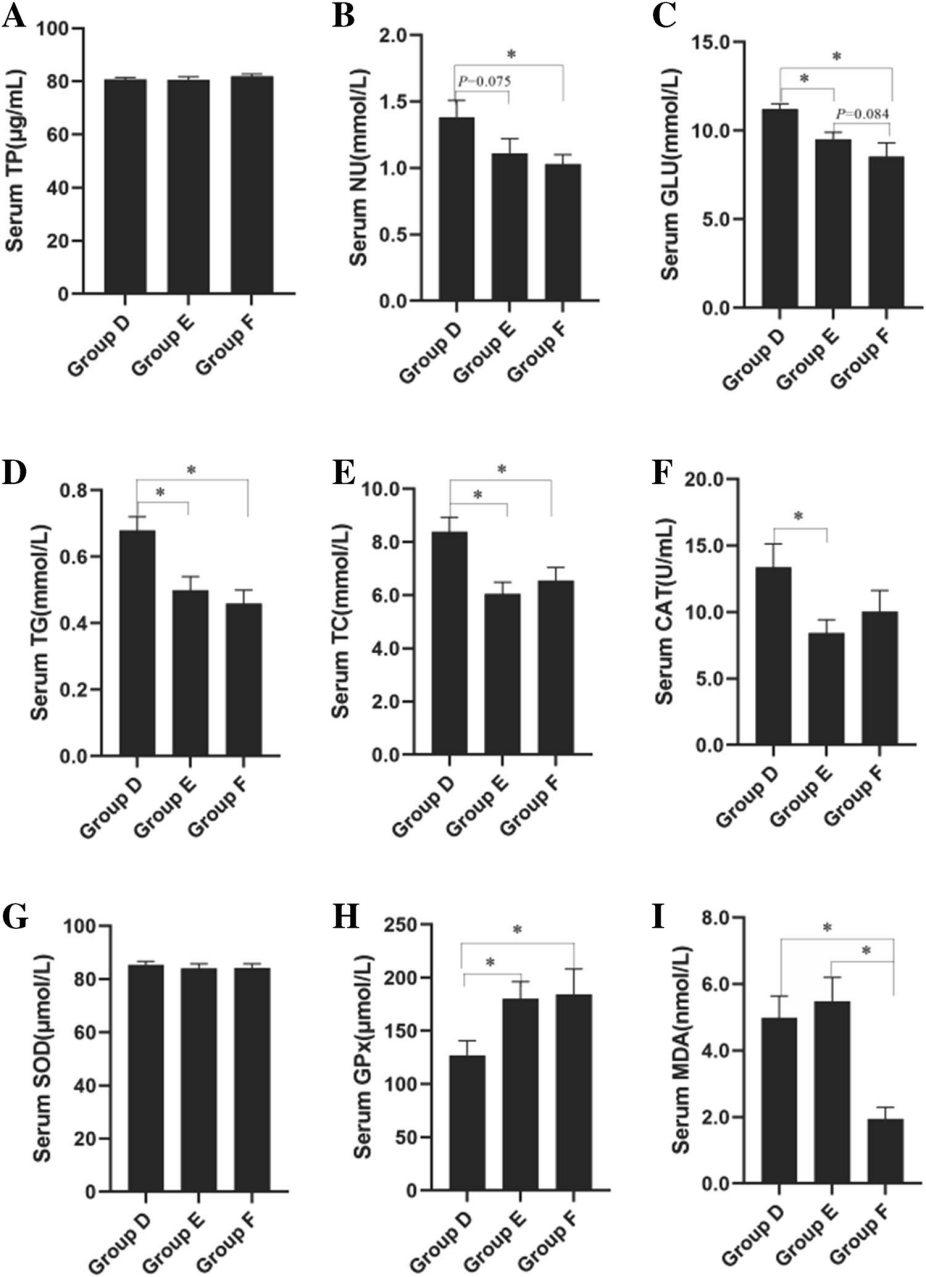
Effects of dietary fiber on serum biochemical indexes of broilers. (**A**) Serum total protein, (**B**) serum urea nitrogen, (**C**) serum glucose, (**D**) serum triglycerides, (**E**) serum total cholesterol, (**F**) serum catalase, (**G**) serum superoxide dismutase, (**H**) serum glutathione peroxidase, (**I**) serum malondialdehyde. Group D: Control group, antibiotic-free basal diet; Group E: basal diet + 5% RB; Group F: basal diet + 1% MBP. Data were presented as Mean ± SD (n = 8). Statistical trend at *P* = 0.05–0.10, “*” indicated *P* < 0.05

At the end of the experiment, the effects of dietary fibers on serum biochemical indicators of broilers are determined (Fig. 2). There is no significant difference in total protein among three groups (*P* > 0.05). Serum urea nitrogen of Group F is significantly lower than that of Group D (*P* = 0.023), while that of Group E tends to be lower than Group D (*P* = 0.075). The levels of serum glucose, triglyceride, and total cholesterol of broilers in Group F and Group E are significantly lower than those of Group D (*P* < 0.05 for all). Serum glucose in Group F tends to be lower than that of in Group E (*P* = 0.084).

The serum catalase activity of broilers in Group E is significantly lower than that of Group D (*P* < 0.05), but there is no significant difference among other groups (*P* > 0.05). Glutathione peroxidase activity of Group F and Group E is significantly higher than that of Group D (*P* < 0.05), while no significant difference is observed between Group F and E (*P* > 0.05). Serum superoxide dismutase activity is not significantly varied among three groups (*P* > 0.05). Serum MDA (malondialdehyde) in Group F is significantly lower than that of Group E and F (*P* < 0.05 for all), while no significant difference is observed between Group F and E (*P* > 0.05).

### The effects of MBP on short-chain fatty acid (SCFA) in cecal chyme of broilers

**Fig. 3:**
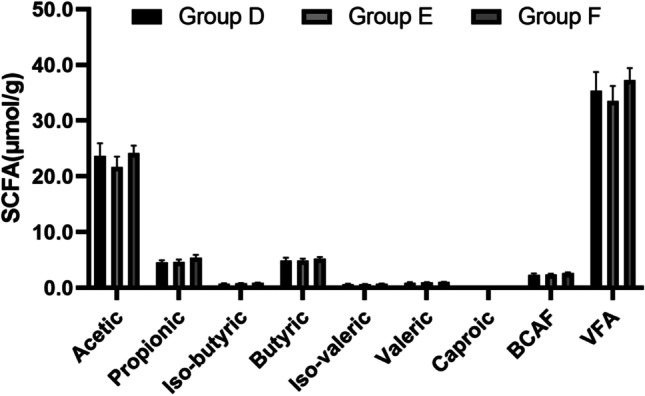
Effects of dietary fibers on SCFA species (μmol/g) in cecal chyme of broilers. BCAF, branched-chain fatty acids = iso-butyric + butyric + iso-valeric. VFA, volatile fatty acids (acetic acid + propionic acid + butyric acid + BCFA). Group D: Control group, antibiotic-free basal diet; Group E: basal diet + 5% RB; Group F: basal diet + 1% MBP. Data were presented as Mean ± SD (n = 8). Statistical trend at *P* = 0.05–0.10, “*” indicated *P* < 0.05

The SCFA species in chyme of broilers are determined with GC–MS, including acetic acid, propionic acid, isobutyric acid, butyric acid, iso-valeric acid, valeric acid, caproic acid, branched chain fatty acid (BCAF) and volatile fatty acid (VFA). There was no significant difference among the three groups in the content of all these SCFA species (Fig. 3, *P* > 0.05 for all), although group F with 1% MBP perfomed best BCAF and VFA.

### The regulation of MBP on the diversity and composition of cecal chyme bacteria in broilers

**Fig. 4:**
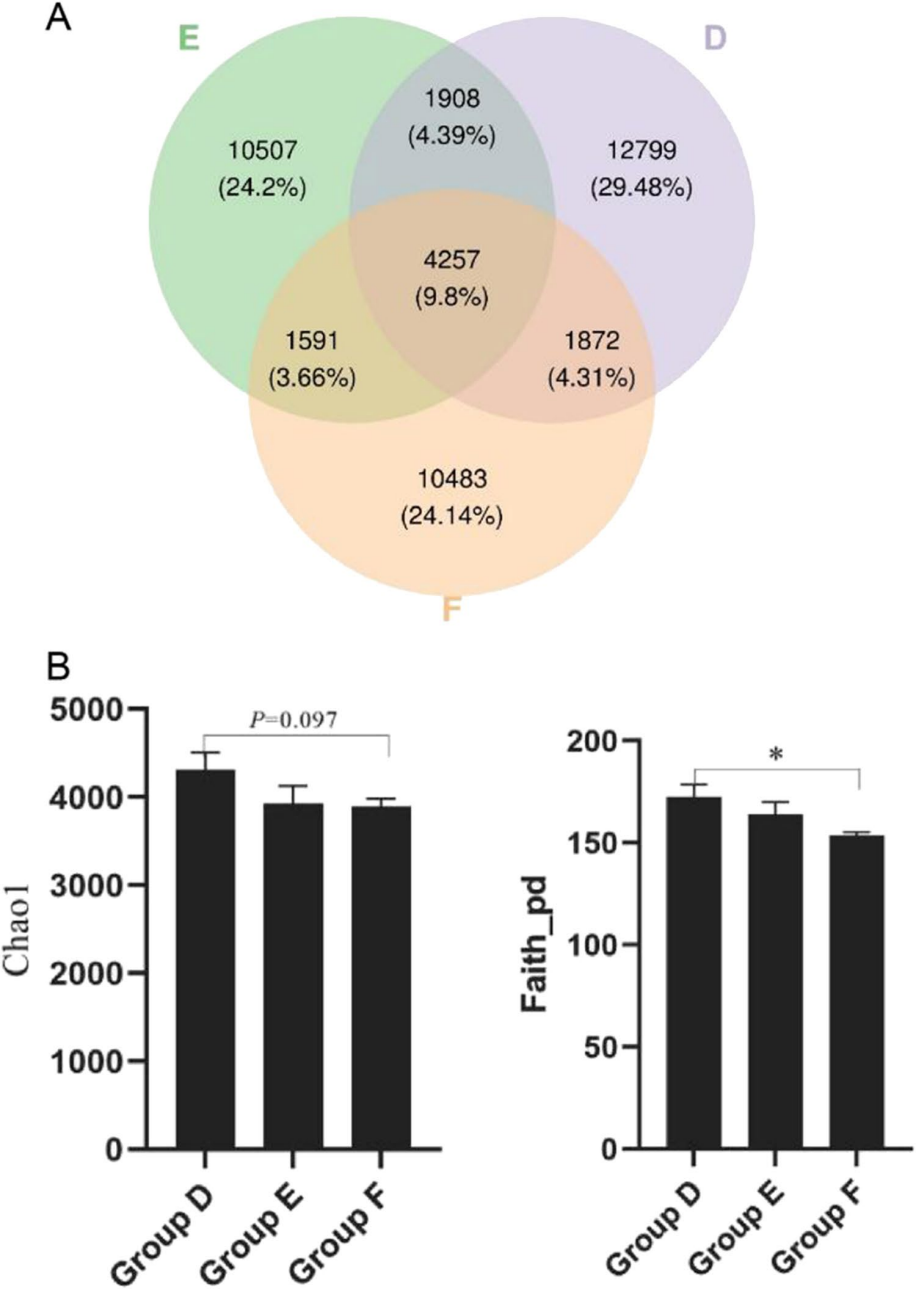
Effects of dietary fibers on microbial abundance and diversity of cecal chyme in broilers. (**A**) ASV/OUT Venn in different groups; (**B**) alpha-diversity in different groups. Group D: Control group, antibiotic-free basal diet; Group E: basal diet + 5% RB; Group F: basal diet + 1% MBP. Data were presented as Mean ± SD (n = 8). Statistical trend at *P* = 0.05–0.10, “*” indicated *P* < 0.05

In order to determine the effects of different fibers on bacterial abundance and diversity in intestinal chyme of broilers, eight fresh cecal chyme samples are collected from each group at the end of the experiment. PCR amplification and Illumina Miseq high-throughput sequencing are performed on the bacterial 16SrRNA V3-V4 region of each sample. A total of 1,841,562 high-quality sequences are used for flora classification, with an average length of 380 bp. The statistical analysis is performed on the flattened ASV/OTU table (Fig. 4). There are 20,836 OTUs in Group D, 18,263 OTUs in Group E, and 18,203 OTUs in Group F (Fig. 4A). Three groups share 4257 OUTs, accounting for 9.8% of the total OUTs. The number of OUTs is the highest in Group D, with 12,799 unique OUTs, accounting for 29.48%, which is relatively higher than that of in Group E and F.

Chao1 is an indicator for microbial characterization abundance and Faith-pd is an indicator for microbial evolution diversity. It can be seen from Fig. 4B, Chao1 of cecal chyme in Group F tends to be lower than that of in Group D (*P* = 0.097). Faith_pd of cecal chyme in Group F is significantly lower than that of Group D (*P* < 0.05). No significant difference is observed among other groups (*P* > 0.05).

**Fig. 5:**
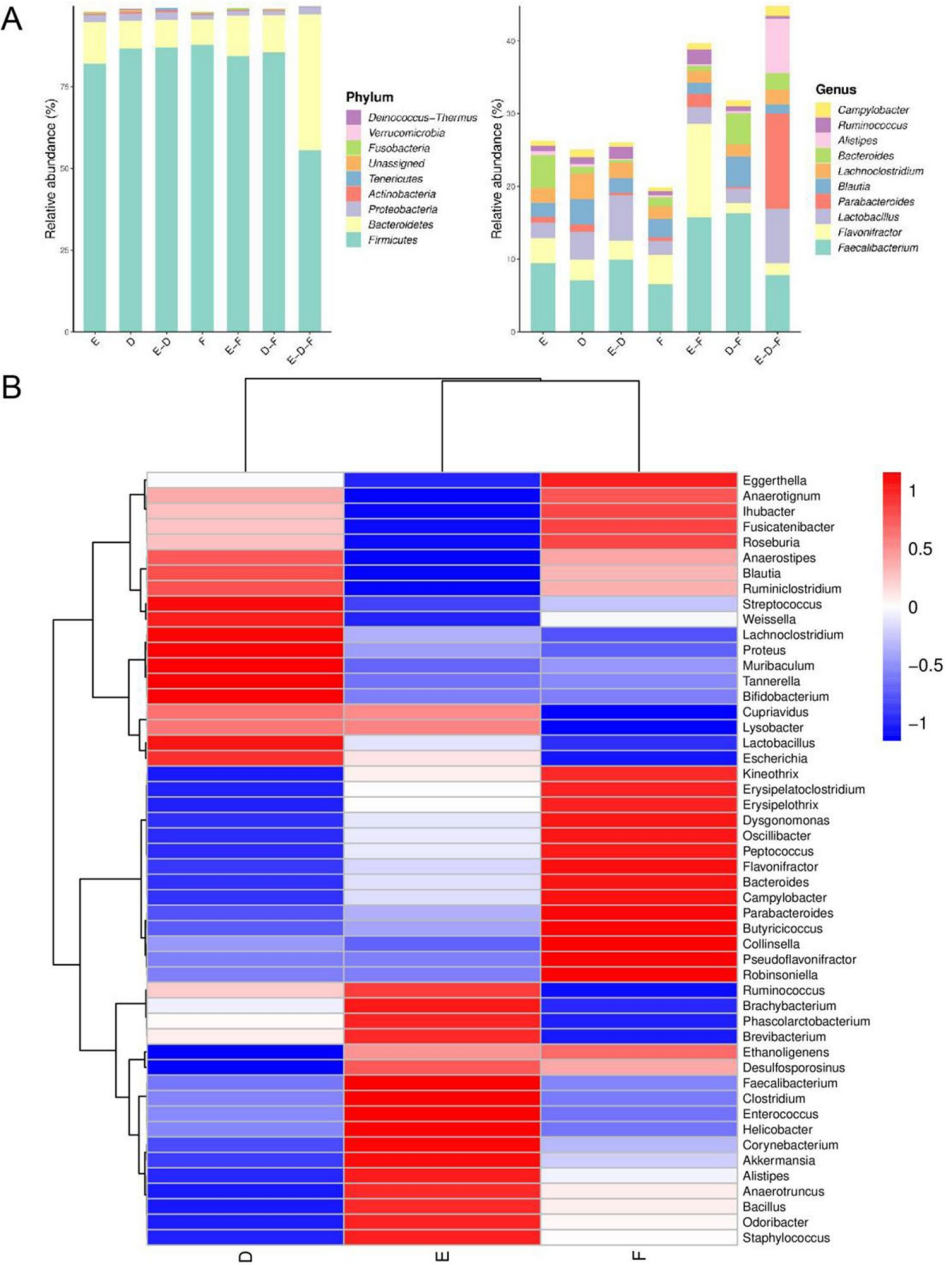
Effects of dietary fiber on cecal microbial composition of broilers. (**A**) ASV/OUT Venn histogram, (**B**) Heatmap of general level species of composition for species clustering. Group D: Control group, antibiotic-free basal diet; Group E: basal diet + 5% RB; Group F: basal diet + 1% MBP. Data were presented as Mean ± SD (n = 8)

The data are aligned in the databases. Combining with the results of OUT species classification, the Venn histogram of ASV/OUT abundance is plotted in different groups at the level of phylum and genus (Fig. 5A), and the heat-map of species composition is plotted in different groups at the level of genus (Fig. 5B). At the phylum level, the cecal chyme of broilers of three groups is dominated by *Firmicutes*, *Bacteroidetes* and *Proteobacteria*, etc. At the genus level, the dominant species are *Faecalibacterium*, *Flavonifractor*, *Lactobacillus*, *Parabacteroides*, *Blautia*, *Lachnoclostridium* and *Bacterodies* in three groups. Compared with Group D, the cecal chyme microflora in Group F and Group E greatly varies (Fig. 5B). The abundance ratio of *Firmicutes* in cecal chyme of broilers in Group F is increased slightly, while Group E presents decreased ratio of *Firmicutes* and increased ratio of *Bacteroides*. Compared with Group D, the abundance ratio of *Flavonifractor* in group F increases, the ratios of both *Faecalibacterium* and *Bacterodies* in Group E are improved.

**Fig. 6:**
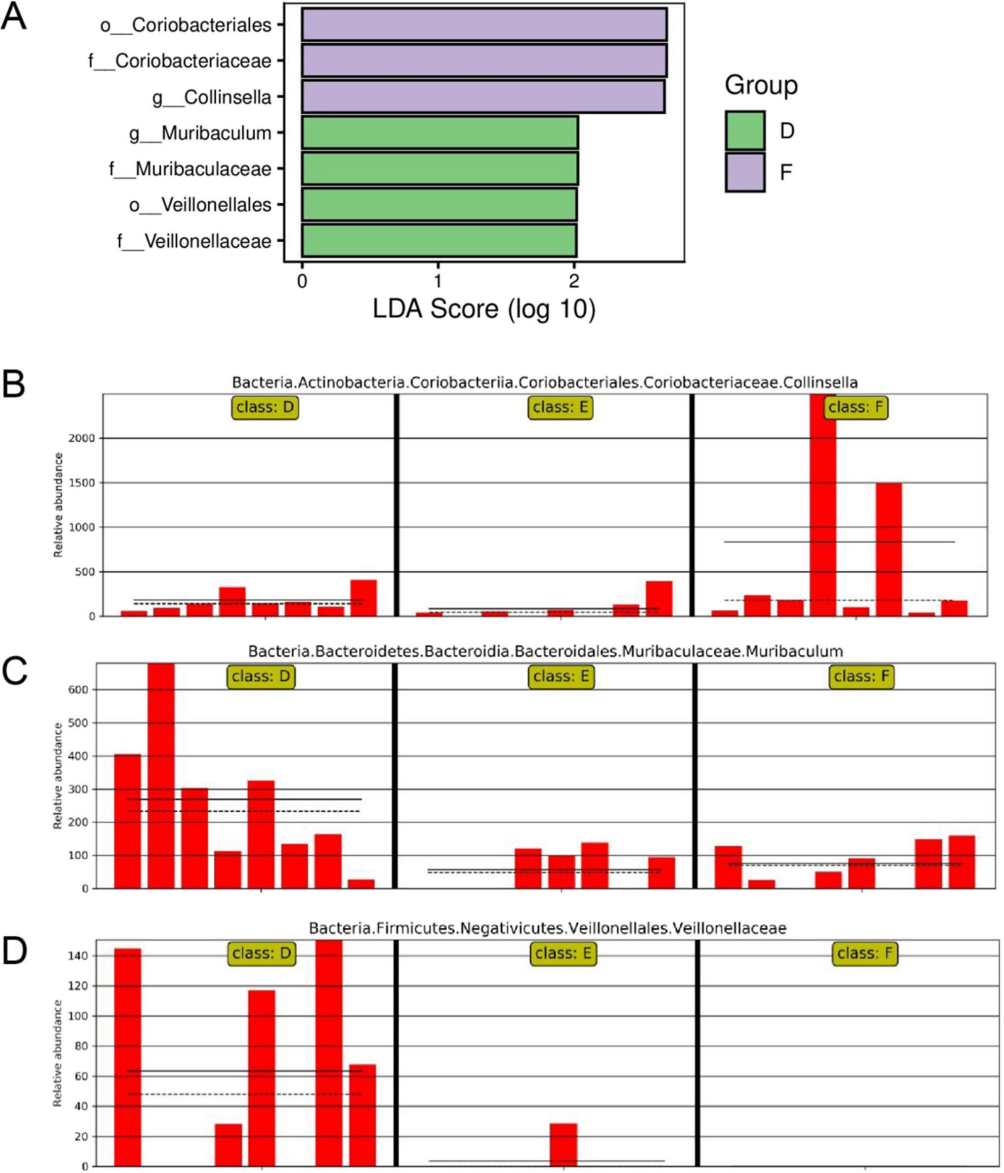
(**A**) Histogram of LDA effect values of marker species. Note: The ordinate represents the taxa with significant differences between groups (*P* < 0.05), while the abscissa visually displays the logarithmic score of LDA analysis for each taxa in a bar chart. (**B**–**D**) The relative abundance distribution of marker species *Collinsella*, *Muribaculum* and *Veillonellaceae* in different groups. Group D: Control group, antibiotic-free basal diet; Group E: basal diet + 5% RB; Group F: basal diet + 1% MBP

By analyzing the difference of flora composition in different groups, the histogram of LDA effect value of marker species can be obtained (Fig. 6A). The marker species shared in three groups are selected (Fig. 6B-D). With LDA analysis, significance difference is observed among the three groups. The abundance of *Collinsella* is high in Group F (Fig. 6B), *Muribaculum* and *Veillonellaceae* are abundant in Group D*.*

### The regulation of MBP on chyme metabolic pathway in broilers

**Fig. 7:**
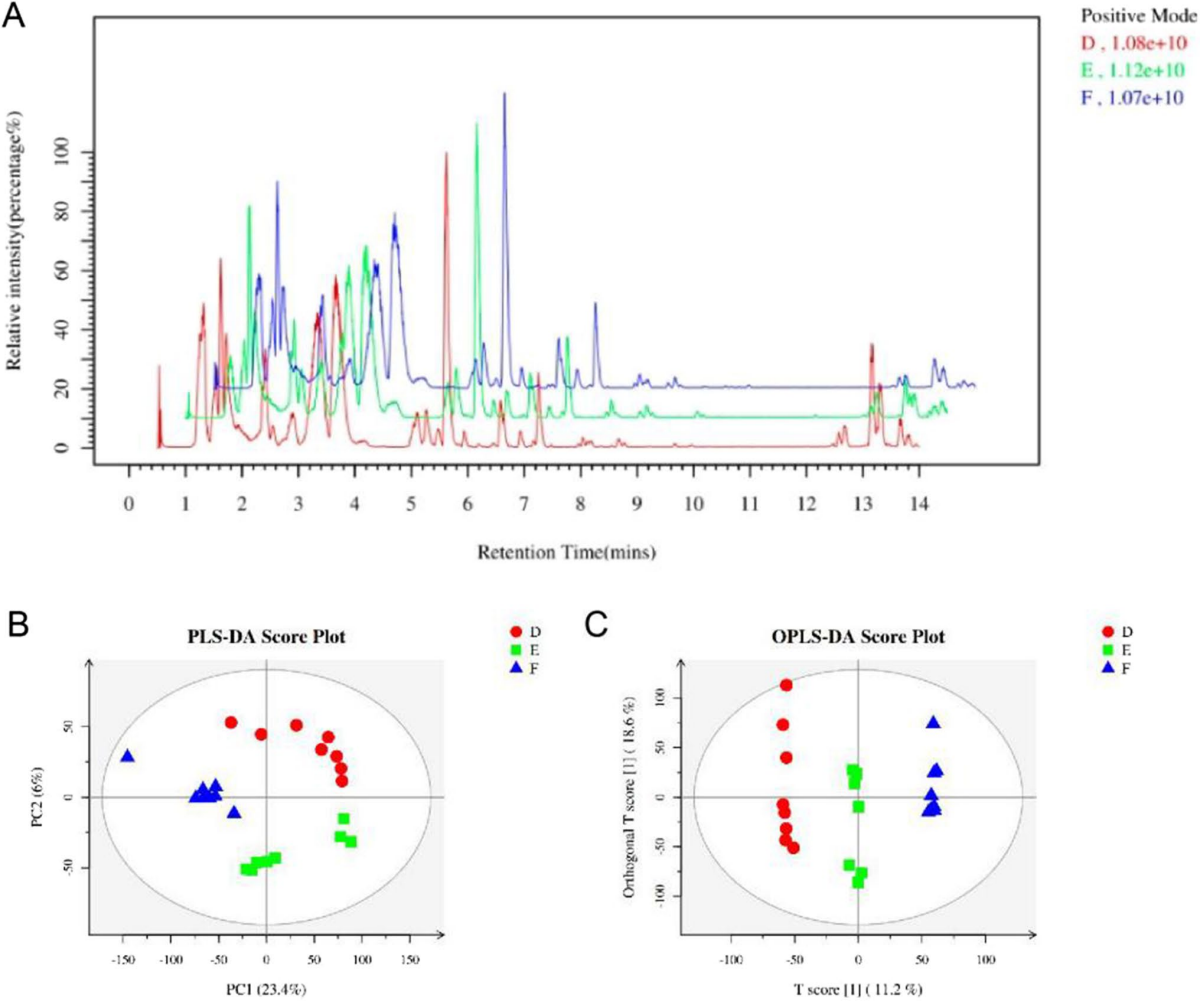
(**A**) Chromatogram of basal peaks of typical cecal chyme samples of broilers in different groups; (**B**) PLS-DA and OPLS-DA scores of cecal chyme of broilers in different groups

The typical chromatograms of cecal chyme of broilers fed with different fibers are analyzed with LC–MS (Fig. 7A). The total ionic strength of cecal chyme metabolites of broilers in three groups varies, including both the metabolite species and their concentrations. The result indicates that the supplement of MBP and RB affects the cecal chyme metabolism of broilers. The detected metabolites are analyzed with Partial Least Squares-Discriminant Analysis (PLS-DA) and Orthogonal Partial Least Squares Discriminant Analysis (OPLS-DA). The sample classification diagram is shown (Fig. 7B). No obvious cross-section and overlap are observed among three groups, indicating significant differences in the chyme metabolites. The regulation effects of different fibers are obvious.

**Fig. 8:**
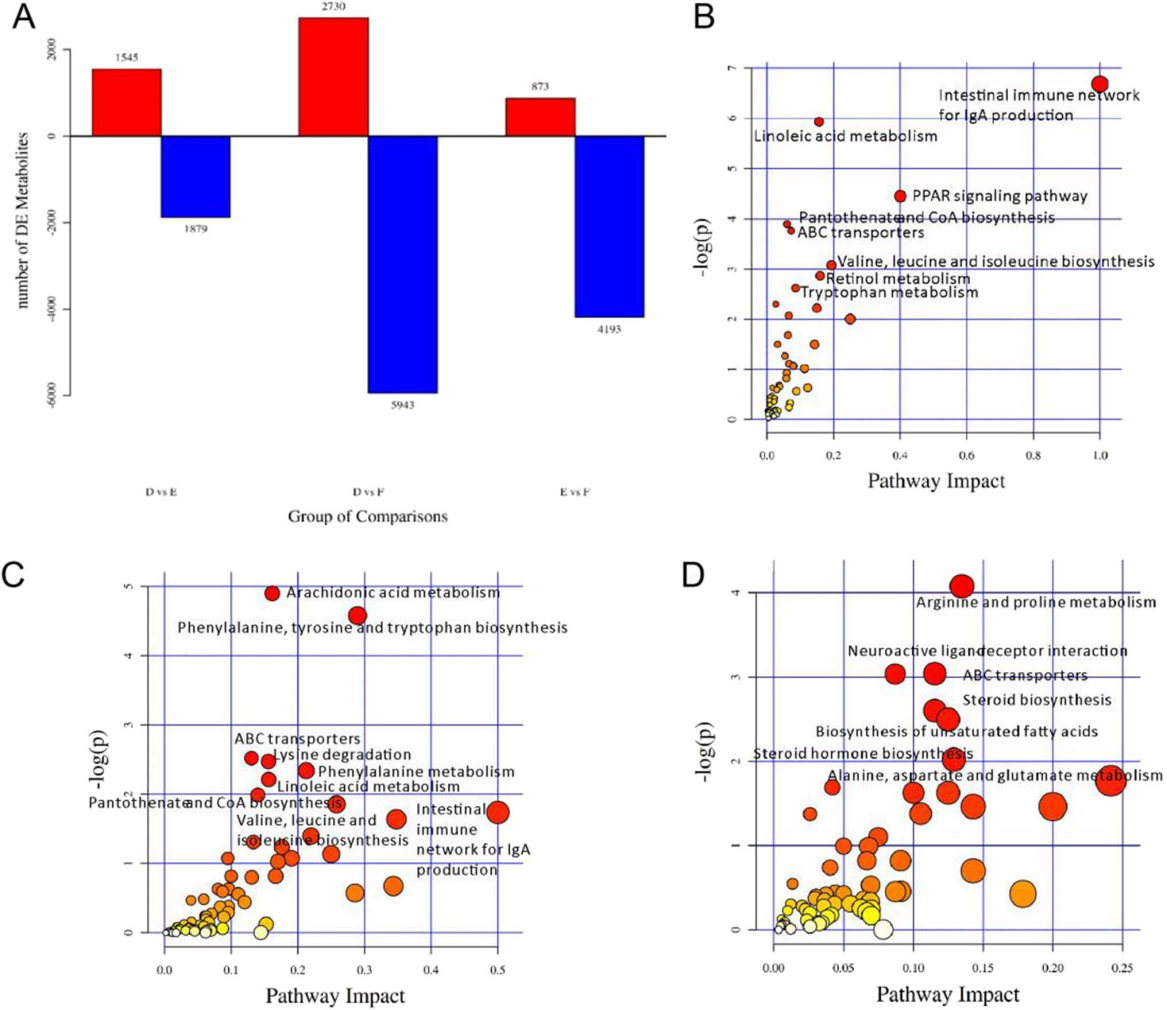
(**A**) Histogram of differential metabolites; (**B**–**D**) metabolome view map of the differential metabolites (VIP > 1, *P* < 0.05) identified in cecal chyme of young broilers between Group D and E (**B**), between Group D and F (**C**), between Group E and F (**D**). The node color is based on its *P*-value, and the node radius is determined based on the pathway impact values. Larger sizes and darker colors represent higher pathway enrichment and impact values, respectively

There are a lot of small molecules in the LC–MS spectra of cecal chyme of broilers. Thus, “1 + 3” conditions are applied for screening the differential metabolites (Fig. 8A). Compared with Group D, there are 1545 up-regulated metabolites and 1879 down-regulated metabolites in Group E, while there are 2730 up-regulated metabolites and 5943 down-regulated metabolites in Group F. Compared with Group E, there are 873 up-regulated metabolites and 4193 down-regulated metabolites in Group F.

The effects of different fibers on metabolism of broilers in cecal chyme are further analyzed. The MetPA database is applied for analyzing the pathways related to the differential metabolites (Fig. 8B). There are 52 different metabolic pathways between Group E and Group D, which are mainly related to intestinal immune IgA production, fatty acid metabolism and amino acid metabolism. 69 different metabolic pathways are observed between Group F and Group D, which are mainly related to fatty acid metabolism, amino acid metabolism and intestinal immune IgA production. A total of 63 different metabolic pathways are found between Group F and Group E, which are mainly related to amino acid metabolism and fatty acid metabolism.

## Discussion

Supplementary of fibers in broiler diets can make beneficial effects on the growth performance, serological indexes and intestinal microbia, etc. For the growth performance, adding 1–1.5% IDF-based cassava pulp modified fiber to broiler diets can reduce abdominal fat deposition, enhance muscle and stomach function and improve nutrient digestibility (Okrathok and Khempaka 2020). Adding 3–6% insoluble fiber to wheat basal diet can improve the growth performance of broilers (Shirzadegan and Taheri 2017). Similar results can be obtained by adding an appropriate level of structural IDF (Jiménez-Moreno et al. 2016). During the maturation and degeneration of lymphoid organs, adding IDF or mixture of IDF and SDF can promote the development of immune system of young birds and chickens (Hussein et al. 2017). However, the digestibility of organic matter and energy was decreased significantly when the level of fibers increased significantly (Röhe et al. 2020). Adding 3–6% insoluble fiber such as alfalfa meal, RB and sawdust in diet had no significant effect on slaughter performance of broilers (Shirzadegan and Taheri 2017). In our study, adding 1% of MBP to broiler diet can improve the G: F and ADG, while it has no significant effect on ADFI, mortality, and most organ indexes. The results indicated that adding MBP to broiler diet was conductive to improving the growth performance of broilers, and the level of 1% was feasible. The dosage could be further tested and optimized from the aspects of organ development and slaughter performance.

In addition, the fiber from different sources also made different effects (Jiménez-Moreno et al. 2016). It was showed that the addition of oat hulls was better than rice husks and sunflower hulls in improving muscle stomach weight, reducing muscle stomach pH and improving nutrient digestibility (Jiménez-Moreno et al. 2019). Our study indicated that the effects of adding MBP in broiler diet were better than that of RB, which may be proved from the G: F and ADG between MBP group and RB group (Fig. 1), and which may be related to the varied physical and chemical properties between the two fibers.

Dietary IDF can affect the serological parameters of broilers. MBP contains 0.94–13.4% of polysaccharide, 1.75–16.89% of starch, 1.31–2.03% of crude protein, and 62.54–89.79% of IDF (Felisberto et al. 2017). It was rich in bamboo leaf flavonoids and polysaccharides, which can regulate the immunity and anti-oxidant capacity of animals (Ge et al. 2020). Bamboo shoot shell fiber had strong cholesterol adsorption activity and prebiotic potential, which can be used as a prebiotic to promote the growth of lactic acid bacteria and increase the fermentability of substrates (Wu et al. 2020). For the mice with hyperlipidemia, it improved the disorder of fat metabolism, reduced the contents of serum cholesterol, triglyceride, low-density lipoprotein cholesterol, and increased the content of high-density lipoprotein cholesterol (Luo et al. 2017). Our study indicated that the addition of MBP regulated the serum biochemical indexes. Compared with RB, the MDA and serum glucose were significantly decreased in broilers fed with MBP. Further, the serum urea nitrogen also tended to be decreased. Chicken can be applied as biological model of growth and development (Vainio and Imhof 1995). The addition of 1.0% IDF-based cassava modified fiber in broiler diet can lower serum cholesterol (Okrathok and Khempaka 2020). It consisted of the results obtained in our study. The addition of fibers from different source can significantly reduce the serum glucose, triglyceride, and total cholesterol in broilers, while significantly increasing the serum glutathione peroxidase activity. The fibers of RB and MBP are mainly made of IDF, indicating that IDF-based fiber material is helpful to improve serum glucose and lipid metabolism of broilers.

The intestinal microbial community has been very important to the host (Ji et al. 2019), which may affect the intestinal function through its metabolites of SCFAs (Clausen and Mortensen 1995), thus making effects on the nutrition, immunity and physiological states of the host (Ussar et al. 2016; Imhann et al. 2018; Rubio 2019). As we all know, cecum can digest some carbohydrates which cannot be digested by small intestine, including cellulose, starch and polysaccharide (Clench and Mathias 1995). Thus, cecal SCFAs were produced by intestinal microorganisms through the fermentation of undigested carbohydrates (Den Besten et al. 2013; Li et al. 2018b). It was found that the variations of fermentation products may be related to the density of microflora (Awad et al. 2016). The highest diversity and abundance of microbial flora have been observed in cecum (Gong et al. 2007; Choi et al. 2014; Liao et al. 2020). After exploring different parts of intestines, it was also found that SCFAs were the most abundant in the cecum (Liao et al. 2020). The difference of SCFAs between small intestine and cecum may be related to the intestinal transportation, intestinal pH and microbial composition (Macfarlane and Macfarlane 2012). Therefore, our study has also focused on SCFAs in the cecum of broilers.

Previous studies have revealed that the SCFAs can significantly affect the intestinal health, which is closely related to the microbials in the intestine. Acetic acid inhibited gastric cell apoptosis and promoted mucin production (Liu et al. 2017), while butyric acid provided energy, maintained the integrity of intestinal epithelial cells and stimulated the growth of intestinal tract (Sun and O'Riordan 2013). It was found that cecal infusion of butyrate stimulated the proliferation of jejunum and ileum cells in piglets (Kien et al. 2007). Butyrate in cecum may be sent back to the small intestine through reverse peristalsis, thus promoting the development of the small intestine. Lactic acid bacteria were positively correlated to acetate in ileum (Liao et al. 2020), which can improve intestinal health by producing some certain SCFAs (He et al. 2019; Zhai et al. 2019). *Salmonella* was negatively correlated with SCFAs including acetic acid, butyric acid and isovaleric acid (Liao et al. 2020), indicating that cecal SCFAs may inhibit the growth and invasion of *Salmonella* (Lawhon et al. 2002).

SCFAs can be changed with the diet. Furthermore, when the level of cellulose was increased, SCFAs decreased significantly (Röhe et al. 2020). In addition, it was found that adding a small amount of fiber in diet of weaned piglets tended to increase the VFA in feces. The amount of lactic acid bacteria and total VFA in feces would be increased significantly under poor sanitary conditions (Mu et al. 2017). In our study, adding 1% MBP had no significant effect on the composition of SCFAs in cecal chyme of broilers (including acetic acid, butyric acid, propionic acid, isobutyric acid, isovaleric acid, valeric acid, etc.), while the levels were increased. It may also be related to the supplementary dosage and culture environment. Further study should be performed to explore whether MBP can promote intestinal health and growth by regulating fermentation and producing SCAFs.

Dietary composition is an important factor affecting intestinal bacteria (Liao et al. 2020). The microbial abundance and diversity in feces could be improved by increasing the levels of dietary fibers, which were indicated by Chao and Shannon indexes (Jiang et al. 2019). It was found that alfalfa diet can increase the diversity of intestinal flora in piglets (Mu et al. 2017). The cecal microbial diversity was rich in birds (Liao et al. 2020). Our study revealed that the addition of MBP significantly reduced the evolution diversity index Faith_pd of cecal chyme microorganism, and tended to reduce the richness index Chao1. The decrease of diversity index is conductive to reducing the nutrient consumption of microbial flora, thus improving the growth performance.

The composition of microflora was complex in gastrointestinal tract of chicken (about 10^7^–10^11^ CFU/g), among which *Firmicutes* were the most abundant, followed by *Proteobacteria* and *Bacteroidetes* (Apajalahti et al. 2004; Sergeant et al. 2014; Xiao et al. 2017). During the growth stage of broilers, the dominant flora in cecum were thick-walled bacteria such as *Faecalibacterium*, *Ruminococcus* and *Lachnospiraceae*, while the dominant flora in cecum were *Bacteroidetes* in the later growth stage. Studies have found that *Ruminococcus* was the main microflora that produced SCFAs in the intestine (Li et al. 2018a), which was positively correlated with the production of butyric acid (Liao et al. 2020). *Trichinella* can promote intestinal development and health by degrading plant fiber and producing SCFAs (Biddle et al. 2013). *Bacteroidetes* can degrade complex carbohydrates by fermenting glucose to synthesize butyric acid as energy of epithelial cells (Macy and Probst 1979), and the abundance of *Bacteroidetes* in cecum was positively correlated with butyric acid concentration (Liao et al. 2020).

The source and structure of dietary fibers can affect the regulation of intestinal flora in broilers. Adding inulin (contained mainly SDF) to broiler diet made greater effects on microflora, however, its effects on weight gain was less than that of inulin and bran combination group (Li et al. 2018a). Adding 2% lignocellulose can reduce *Clostridium*, without affecting the amount of *Bifidobacterium*, *Bacteroides*, *Bacillus* and *Lactobacillus* (Kheravii et al. 2017). It has been found that amorphous cellulose with IDF as the main component can change the composition of microflora at the level of *Bacteroidea* in the chyme, especially *Alistipes* (De Maesschalck et al. 2019). However, when the level of cellulose increased significantly, *Shigella chymotryi* decreased significantly (Röhe et al. 2020). In this study, the addition of MBP tended to increase the richness ratio of *Firmicutes* and *Flavonifractor* in cecal chyme of broilers, while the addition of RB decreased the ratio of *Firmicutes* and increase the ratio of *Bacteroides*, the abundance ratio of *Festuca* and *Bacteroides* was also increased. Further results indicated that the addition of MBP made great effects on cecal chyme flora of broilers, in which the abundance of *Muribaculum* and *Veillonellaceae* was significantly reduced, and the abundance of *Collinsella* was significantly increased. It indicated that MBP can not only regulate the richness and diversity of microflora, but also affect their composition.

Similar results can be obtained by typical chromatogram analysis and metabolic component cluster analysis on the cecal chyme of broilers. The addition of MBP and RB can both affect the total ionic strength and component clustering of cecal chyme metabolites in broilers. It indicated that the addition of IDF can change the species and concentration of metabolites, thus affecting the cecal chyme metabolism. It was also confirmed by the further analysis of metabolic pathways. Compared with the control, the differential metabolic pathways after the addition of MBP were mainly fatty acid metabolism, amino acid metabolism, and intestinal immune IgA production. In addition, the effects of adding different IDF on metabolic pathways also varied. The differences between adding MBP and RB were mainly pathways responsible for amino acid metabolism and fatty acid metabolism.

Fibers play the role of prebiotics in cecal digestion of animals, promoting the selection of healthy intestinal microflora (Kheravii et al. 2017; Donadelli et al. 2019). It is still necessary to further explore the mechanism on the effects of adding MBP in broiler diet. The analysis of differential metabolic pathway confirmed the effects of MBP. Further study can be focused on the fatty acid metabolism, amino acid metabolism, and intestinal immune IgA production.

## Conclusion

Bamboo powder is a kind of abundant fiber resource, which may be used as a prebiotic to assist animal cecal digestion and promote the formation of healthy intestinal microflora. This study revealed that 1% MBP could improve the growth performance of broilers in antibiotic-free basal diet, which is better than that of 5% RB. This may be related to the differences of these two fiber raw materials in regulating serum antioxidant capacity, chyme microbial composition, diversity and metabolic pathways.

## Data Availability

The datasets used or analyzed during the current study are available from the corresponding author on reasonable request.

## Code availability

The software SPSS25.0, QIIME2(2019.4), and Greengenes database in the current study were open to researchers around the world.
